# Chromium-for-Aluminum Substitution in Synthetic Serpentine

**DOI:** 10.3390/nano16080448

**Published:** 2026-04-09

**Authors:** Huang Lin, Hui Zhang, Benjamin Gilbert

**Affiliations:** 1 School of Emergent Soft Matter, South China University of Technology, Guangzhou 511442, China; molinhuang@mail.scut.edu.cn; 2 Center for Electron Microscopy, South China University of Technology, Guangzhou 511442, China; 3 Energy Geoscience Division, Lawrence Berkeley National Laboratory, Berkeley, CA 94720, USA; 4 Department of Earth and Planetary Science, University of California, Berkeley, CA 94720, USA

**Keywords:** hydrothermal synthesis, Cr substitution, Al-lizardite, amesite, serpentine

## Abstract

Cr-bearing clay minerals are products of hydrothermal alteration and fluid–rock interactions of ultramafic rocks that form serpentine minerals. Cr is typically observed to substitute for Al in serpentine minerals, but the crystal chemistry and environmental constraints on this substitution are unknown. Here, we synthesized endmember and Cr-substituted amesite, a typical Al-serpentine mineral, via the hydrothermal method. We found that the phase purity highly depends on the pH of the hydrothermal solution, which should be controlled at ~12.7 to avoid the formation of impurity phases. Additionally, amesite can incorporate Cr at a concentration equivalent to ~39.5% substitution of Al. The Cr-free and Cr-substituted amesite are highly defective and contain multiple polytypes, including 6R_2_, 2M_1_, and possibly 2H_2_. However, the relative proportions of these polytypes do not change with increasing chromium substitution.

## 1. Introduction

Hydrous layered aluminosilicates, also known as clay minerals, possess strongly anisotropic layered structures, which confer fascinating physicochemical properties [1,2,3,4] and vast civil and industrial applications [1,5,6,7]. They are abundant and usually fine-grained in soils and sedimentary rocks. Many phases can be synthesized via hydrothermal reactions with the composition and particle morphology being well-controlled, which has boosted the ever-increasing interest in nano-clay materials for applications such as energy storage, environmental protection and chemical catalysis [1,5,6,8,9,10,11,12,13,14].

Clay minerals are formed by the stacking of silicon–oxygen tetrahedral (T) sheets and metal oxide octahedral (O) sheets, typically in a 1:1 or 2:1 ratio [4]. Isomorphous substitution of metal ions within either sheet is a ubiquitous crystal chemistry feature of clay minerals, which affects many important properties. Al-for-Si substitution is common in serpentine, a family of 1:1 tri-octahedral clay minerals. Studies of natural serpentines have shown that the substitution extent varies with alternative layer stacking structures or polytypes. For lizardite, the most common serpentine with the ideal formula Mg_3_Si_2_O_5_(OH)_4_, the 1T or 2H_1_ polytypes contain very little Al (~3% octahedral sites) [15] while the 2H_2_ polytype may have 17.3% Al in O-sheets and 29.5% Al in T-sheets [16]. Higher Al concentrations favor the formation of amesite, (Mg_2/3_Al_1/3_)_3_(Si_1/2_Al_1/2_)_2_O_5_(OH)_4_, in which Al substitutions are fully or partially ordered within the O-sheets (up to 33.3% Al) and T-sheets (up to 50% Al) [17,18,19,20,21].

Clay mineral sites that can incorporate Al can also host other trivalent metal ions, including chromium. Clay minerals are an important reservoir of Cr in serpentine soils [22,23]; however, the structural controls on the limits of Cr substitution and control on Cr distribution are very challenging to establish from natural clays. It has been demonstrated that 18% of octahedral cations in montmorillonite and 8.5% in halloysite are Cr [24] in the rocks from the Crommyonia volcanic area (Greece). In contrast, Cr in kaolinite from Teslić (Republika Srpska) is quite limited, only ~1.5% [25], whereas the Cr substitution of Al can be 8.5% in illite [26], and reaches 31.5% in smectite [27]. In our recent study of natural serpentine minerals, the Cr concentration at the octahedral sites reached only a few percent [21]

As it is difficult to determine the Cr substitution limit from studies of natural clay minerals, we sought to use the hydrothermal synthesis to study Cr incorporation in amesite. Here, amesite was chosen because it has the highest Al concentration in the serpentine family and thus can provide a wide range in Cr-for-Al substitution. The hydrothermal synthesis of clay minerals has been extensively explored [6,28,29,30,31], with the synthesis strategy mainly differing in the use of gels as precursors or not [31]. In a recent synthesis effort, Al-lizardite was hydrothermally synthesized at 200 °C with intensely ground kaolinite and hydroxide powders [32]. However, in these synthesis methods, 25% Mg-containing kaolinite was always present as an impurity phase, and the Al substitutions at O and T sites were only 26.4% and 13%, respectively. It is expected to be more challenging to synthesize the high Al concentration counterpart of lizardite, amesite, by this method. Accordingly, here we used the gel-making method to synthesize endmember and Cr-substituted amesite.

## 2. Methods

### 2.1. Gel Preparation

Amesite nanopowders were synthesized via the gel-making route [33], where tetraethyl orthosilicate, Si(OC_2_H_5_)_4_ (Aldrich, reagent grade) and aluminum isopropoxide, Al(OCH(CH_3_)_2_)_3_ (Aldrich, reagent grade) served as the Si and Al source, respectively. MgCl_2_·6H_2_O (Aldrich, reagent grade) and CrCl_3_·6H_2_O (Aldrich, reagent grade) were chosen as the Mg and Cr sources. The molar ratios of Si(OC_2_H_5_)_4_:Al(OCH(CH_3_)_2_)_3_:MgCl_2_·6H_2_O:CrCl_3_·6H_2_O are 1:(2 − 3*x*):2:3*x*, where *x* = 0, 0.1, 0.2, 0.33. These are referred to as 0Cr, 10Cr, 20Cr and 33Cr, respectively.

First, MgCl_2_·6H_2_O (4.066 g; 0.02 mol) and the desired quantity of CrCl_3_·6H_2_O (0 g, 0 mol; 0.7993 g, 0.003 mol; 1.599 g, 0.006 mol; 2.638 g, 0.099 mol) were dissolved in deionized water (150 mL) with continuous magnetic stirring. Then, 2.230 mL tetraethyl orthosilicate was pipetted into the mixture solution. After that, the corresponding amount of aluminum isopropoxide powder was slowly added. This process was repeated 3~5 times to obtain the desired quantity. After the final addition, the mixture was stirred for 24 h to form gels. All procedures were performed under ambient conditions. Finally, the gels were dried in an air oven at 60 °C for 12 h and then powdered and stored in a desiccator.

### 2.2. Hydrothermal Syntheses

Either 0.8 g or 1 g of the dried gel powders were weighed into 15 mL liquid solutions (deionized water and 1 M KOH) and then mixed with magnetic stirring for 1 h. After that, the mixture solution was poured into a 50 mL Teflon-lined reactor. The amount of KOH was controlled to adjust the pH of the liquid, which was measured by a pH meter. Only 0.8 g of powdered gel was used for 20Cr and 33Cr samples to avoid the formation of impurity phases. The assembled reaction vessels were put in the oven, kept at 250 °C for one week, and then air-cooled to room temperature. After removing the liquid, the final products were washed by centrifugation five times to remove KCl and other soluble materials. Finally, the products were dried, powdered, and stored in a desiccator.

### 2.3. Characterizations

To determine the phase components of the synthesized powders, X-ray diffraction (XRD) patterns were collected on a Rigaku SmartLab 9 kW diffractometer with Cu Kα radiation. The step size was 0.02°. Attenuated total reflection Fourier transform infrared spectroscopy (FTIR, Nicolet 6700, Thermo Fisher Scientific, USA) was used for the spectroscopic study. The compositions were determined by inductively coupled plasma mass spectrometry. Transmission electron microscopy (TEM) was used for the microstructural characterization. For cross-sectional observation, the synthetic powders were embedded in M-bond (Ted Pella, USA), which was sandwiched between two glass slides, and then solidified. Then, cross-section samples were cut and polished and finally made electron transparent by ion milling. The TEM studies were performed on the Thermo Fisher Themis working at 200 kV. The electron energy loss spectra (EELS) were collected with a K3 camera to study the valence status of Cr.

## 3. Results

**Figure 1:**
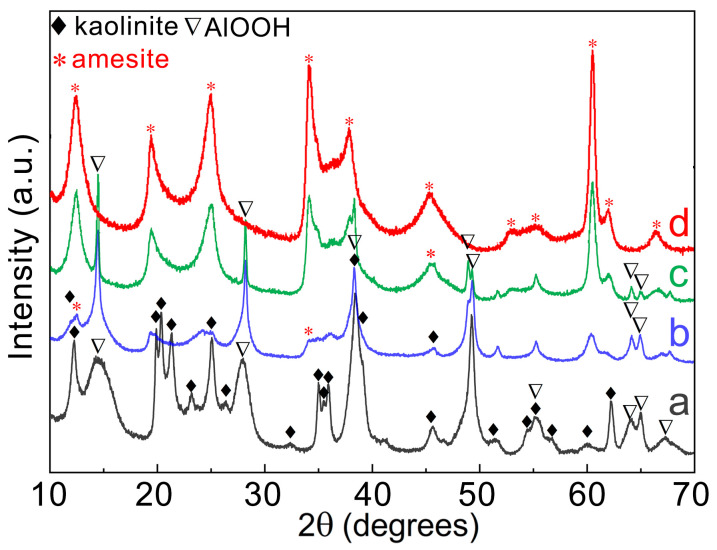
XRD patterns of the samples synthesized under different pH conditions: (**a**) pH = 6.32, (**b**) pH = 9.84, (**c**) pH = 12.53, and (**d**) pH = 12.72. The synthesis conditions and final phase components are listed in Table 1. Phase-pure amesite within the detection limitations of XRD was obtained under condition (**d**).

As a typical dioctahedral 1:1 clay mineral, kaolinite can be synthesized in a wide pH range from acid to base [31,33] via the hydrothermal method. However, for the trioctahedral 1:1 clay mineral amesite, we found that pH had a crucial role in the products. To elucidate the effect of pH on the formation of amesite, the pH values of the starting mixture were tuned by changing the relative volume of deionized water and 1 M KOH solution. Figure 1 shows the XRD patterns, and Table 1 lists the conditions and corresponding final phases. For sample 0Cr-a, the final products contain a large amount of boehmite besides the kaolinite, but no peaks related to amesite could be observed, as shown in Figure 1a. Increasing the pH to 9.84 significantly suppresses the formation of kaolinite, and boehmite dominates, with minor amesite being formed (Figure 1b). A further increase in pH promotes the formation of amesite (Figure 1c), and synthesis with a pH of 12.72 produces phase-pure amesite within the detection limitations of XRD (Figure 1d).

**Figure 2:**
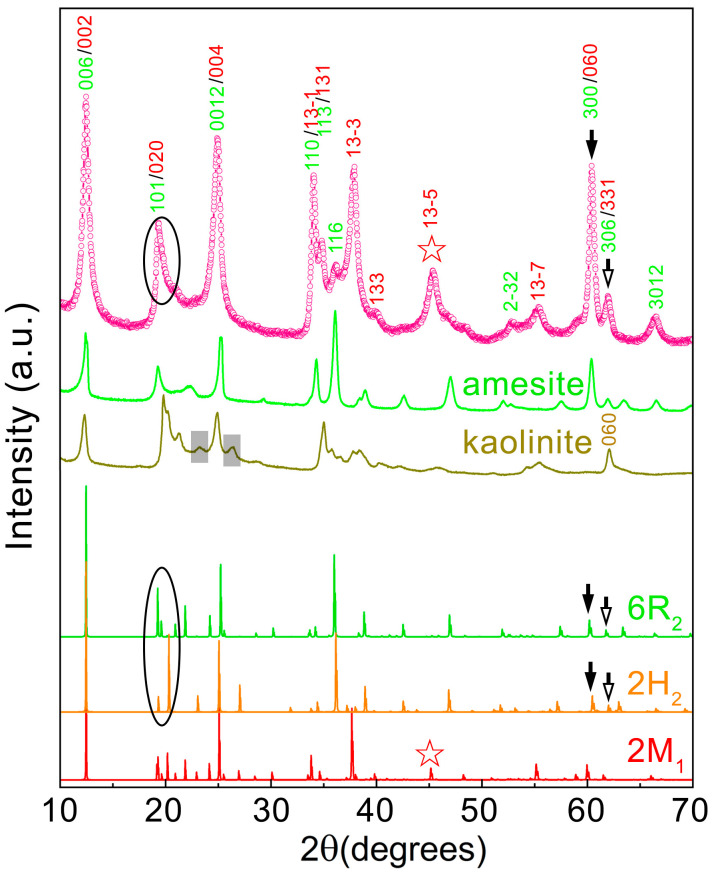
Experimental XRD patterns of 0Cr (pink) prepared with the 0Cr-d condition listed in Table 1: natural amesite (green), kaolinite (brown), and simulated XRD patterns of amesite with 6R_2_, 2H_2_ and 2M_1_ polytypes. The stars denote the fingerprint peak of 2M_1_. Solid and hollow arrows denote the peaks usually designated to the (060) diffraction of trioctahedral (amesite) and dioctahedral (kaolinite) 1:1 clay minerals when an orthorhombic unit cell is used, respectively. Our analyses show that these peaks come from amesite only. Red and green indices denote the peaks of 2M_1_ and 6R_2_, which have orthorhombic and hexagonal unit cells, respectively. For 6R_2_, the Bravais–Miller index was used with the third index omitted.

The XRD data in Figure 2 indicate that the synthetic amesite contains multiple polytypes, in common with other synthetic clay minerals [34]. The XRD pattern of 0Cr bears a great resemblance to that of natural amesite from the Saranovskoye mine (Russia) shown in the green curve. Prior work demonstrated that this natural semi-disordered amesite is mainly composed of the 6R_2_ and 2H_2_ polytypes [21]. The 6R_2_ polytype is more dominant than 2H_2_, even though 2H_2_ is reported to be the most abundant in fully ordered amesite crystals [17,35]. To establish the most likely polytypes in 0Cr, the XRD patterns of twelve standard polytypes and nonstandard polytype 6R_2_ [36] were calculated and compared with the experimental pattern from synthetic amesite. The peaks marked by arrows near 60° are indicative of 6R_2_ and 2H_2_. Comparing the circled peaks between experimental and simulated patterns suggests that 6R_2_ is also more dominant than 2H_2_ in 0Cr. However, a distinct reflection was observed at 2θ ≈ 45°, as indicated by the star, which is absent in the pattern of the natural amesite and is indicative of a different polytype. The peak at 2θ ≈ 45° is diagnostic for 2M_1_ and absent in 6R_2_ and 2H_2_. Thus, the XRD data from synthetic amesite can be fully indexed using 6R_2_ and 2M_1_ polytypes. The XRD data do not completely rule out the existence of the 2H_2_ polytype but strongly indicate 6R_2_ to dominate. The crystal structure information of these polytypes could befound in Bailey’s paper [36].

**Figure 3:**
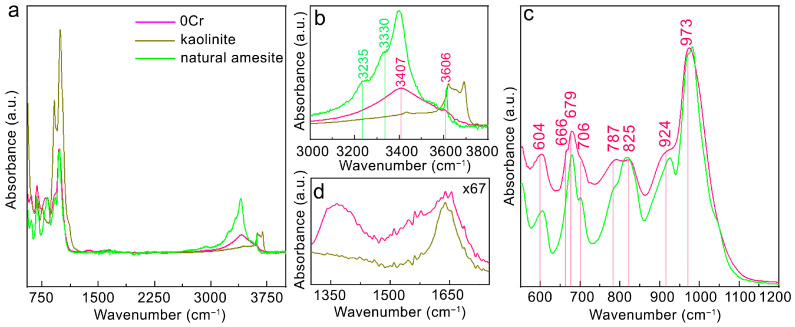
(**a**) FTIR spectra of 0Cr, natural amesite and synthetic kaolinite. (**b**) The high wavenumber region, where the significant contrast between synthetic amesite and kaolinite suggests that kaolinite is absent in 0Cr. The peaks in the middle wavenumber range are labeled in (**c**). (**d**) Weak peaks in 1300~1750 cm^−1^. The intensities were scaled by a factor of 67 for clarity.

A common issue for the hydrothermal synthesis of sheet serpentine is that kaolinite often exists as an impurity phase [32,34]. The peak marked by the hollow arrow in the pattern from synthetic amesite coincides with the (060) peak of kaolinite. However, other kaolinite reflections are not observed. In addition, the kaolinite (060) peak can also be designated as (306) of 6R_2_ and (331) of 2M_1_. Therefore, XRD alone cannot conclusively determine if there is a small amount of kaolinite. FTIR was performed to further assess the kaolinite content. As shown in Figure 3a, the spectrum of 0Cr significantly differs from that of kaolinite, especially in the high wavenumber region (Figure 3b). Thus, we conclude that 0Cr does not contain kaolinite and is pure-phase amesite within the detection limitations of XRD.

A complete assignment of the FTIR spectra has not been reported in amesite so far; hence, here we refer to the work on lizardite [32,37,38,39] and compare it with the spectrum of natural amesite for a detailed analysis. Figure 3c displays the medium range spectrum, where the absorption bands of 0Cr and natural amesite are nearly identical. The band at 604 cm^−1^ is unique for serpentine minerals [32,37] and is attributed to the in-plane movement of hydrogen atoms [38]. The bands at 666 cm^−1^, 679 cm^−1^, 706 cm^−1^, 787 cm^−1^, 825 cm^−1^, 924 cm^−1^ and 973 cm^−1^ are attributed to Si–O vibrations in the tetrahedra. For example, the 924 cm^−1^ and 973 cm^−1^ bands are related to the equatorial and symmetric stretching modes of Si–O, respectively [38]. In addition to those main vibrational bands, weak signals at 1364 cm^−1^ and 1643 cm^−1^ (Figure 3d), attributable to carbonates [40] and adsorbed water [41], were observed in the spectra of synthetic amesite and kaolinite.

Clear distinctions between 0Cr and natural amesite could be observed in 3000~3800 cm^−1^. For 0Cr, there is only one broad band centered at 3407 cm^−1^ and a tiny shoulder peak at 3606 cm^−1^. For natural amesite, two shoulder peaks, at 3235 cm^−1^ and 3330 cm^−1^, before the main peaks at 3397 cm^−1^ and 3615 cm^−1^ were identified. All these bands are attributed to the stretching of hydroxyl groups present on the outer surface or between adjacent TO layers (Table 2) [37,38,39]. The broader bands in 0Cr compared to natural amesite suggest great disorder in synthetic amesite, especially for the bonding environment of interlayer hydroxyl groups.

**Figure 4:**
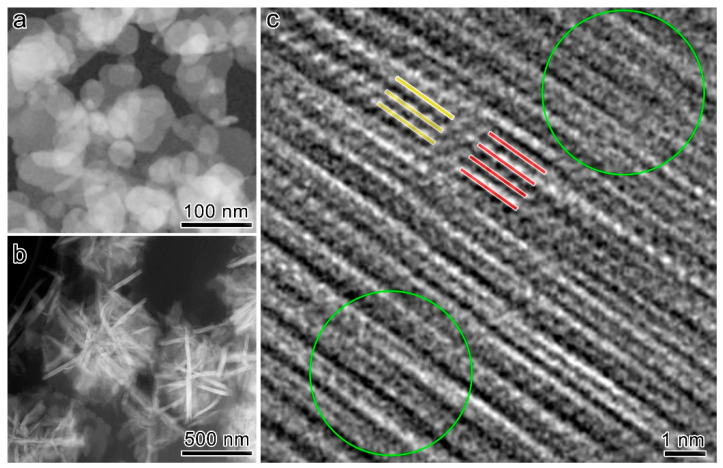
(**a**,**b**) HAADF–STEM plane-view (**a**) and cross-section (**b**) morphology of 0Cr. (**c**) High-resolution TEM image. Green circled regions only show faint lattice contrast because of large orientation deviation. Red and yellow lines highlight regions of interrupted basal planes.

The amesite particles have a plate-like morphology. Figure 4a,b show the HAADF–STEM images of 0Cr in plane-view and cross-section samples, where the lamellar particles are prone to lying face-on and edge-on, respectively. The platy particles are, by and large, equiaxial, and have a nominal lateral size of 50~100 nm and a thickness of 10~20 nm. High-resolution TEM provides direct evidence of a highly defective stacking structure (Figure 4c). The orientation alters dramatically even within a ~10 nm field of view, such that the green circle regions are not atomically resolved and only show faint lattice fringes. For the on-zone regions, interrupted basal planes are ubiquitously observed (red and yellow lines). The basal planes marked by yellow lines are shifted along the direction perpendicular to the basal planes relative to the basal planes marked by red lines, which resembles the basal plane shift in antigorite [42].

**Figure 5:**
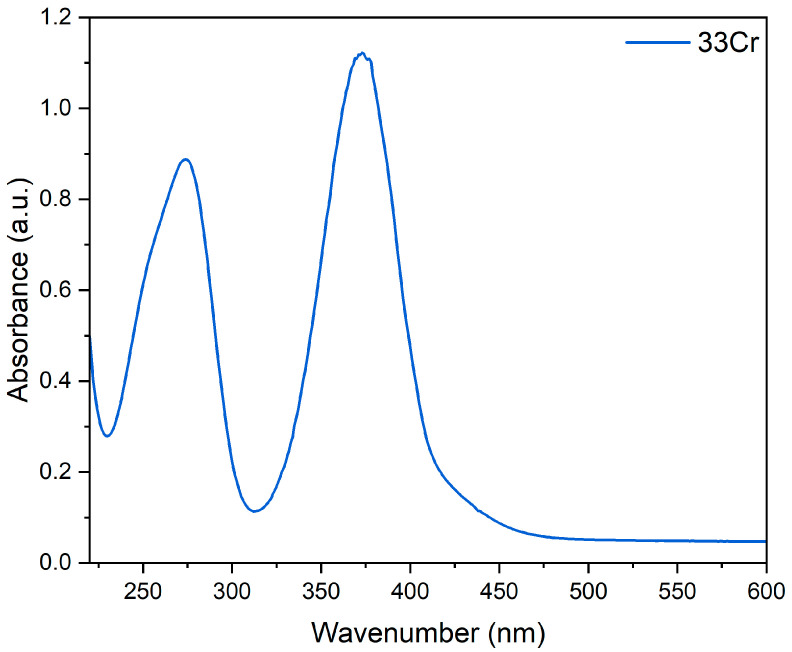
UV-Vis spectrum of the supernatant of 33Cr.

By controlling the pH at around 12.7, three Cr-containing amesite samples, 10Cr (*x* = 0.1), 20Cr (*x* = 0.2), 33Cr (*x* = 0.33), were synthesized (Table 3). In 10Cr and 20Cr, ~14.0% and ~27.5% Al are replaced by Cr, which are close to the designed concentrations 15.0% and 30.0%, respectively. Nevertheless, only ~39.5% Al is substituted by Cr in 33Cr, which is significantly lower than the designed 49.5%, indicating that the substitution limit is around ~39.5%. The UV-Vis spectrum of the supernatant shows two peaks at ~280 nm and ~370 nm (Figure 5), indicating that the remaining Cr^3+^ in the precursor was oxidized to Cr^6+^ and subsequently partitioned into chromate in the supernatant [43]. Trace impurities from the starting chemicals, Ca, Na, K and Fe were identified in the samples. The amesite particles in 10Cr, 20 Cr and 33Cr also show plate-like morphologies, and the particle sizes are similar to those in 0Cr. The defective microstructures shown in Figure 4 were also observed.

**Figure 6:**
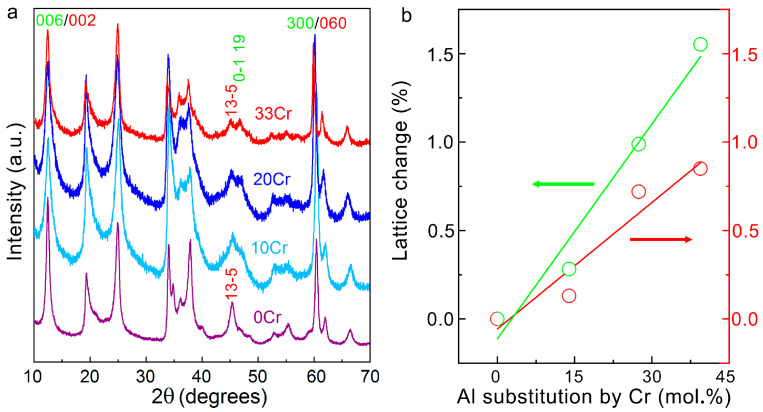
(**a**) Comparison of the XRD patterns of 0Cr, 10Cr, 20Cr and 33Cr. (**b**) Lattice change versus Al substitution by Cr. Green and red circles denote the relative change in *c*- and *a*-lattice-parameters, respectively. Fitted lines are plotted to illustrate the trends.

**Figure 7:**
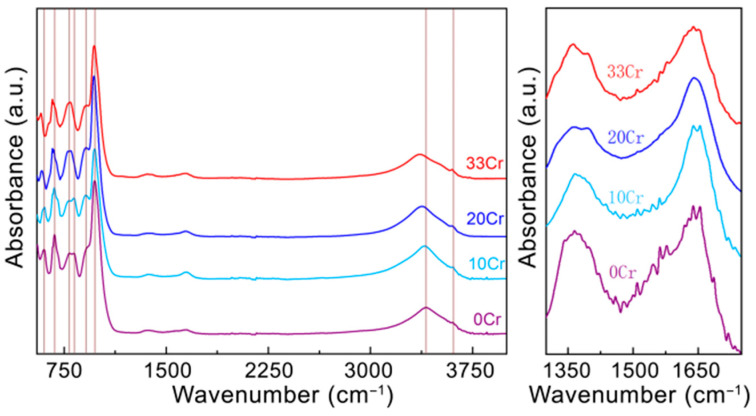
FTIR spectra of 0Cr, 10Cr, 20Cr and 33Cr amesite. Most peaks exhibit redshifts within increasing Cr content. The weak peaks between 1300 and ~1750 cm^−1^ can possibly be caused by carbonates and residual water, as presented in the right column.

As can be seen in the XRD data in Figure 6a, the peaks of 10Cr, 20Cr and 33Cr are basically the same as those of 0Cr, and polytypes should be 6R_2_, 2M_1,_ and possibly 2H_2_ as well. No impurity phases can be identified in the XRD patterns. Interestingly, the $(13\overset{-}{5})$ peak in 0Cr splits into two peaks in 20Cr and 33Cr, designated as $\left(13\overset{-}{5}\right)$ of 2M_1_ and $\left(0\overset{-}{1}19\right)$ of 6R_2_, suggesting that the coherency of the two polytypes deteriorates as the Cr substitution mounts. As Cr substitution increases, the *c*- and *a*-lattice-parameters also increase gradually (Figure 6b). The trend in lattice dimension is accompanied by a redshift of the absorption bands in the FTIR spectra (Figure 7). This shift is attributed to the increasing substitution of heavier Cr for Al, which does not, however, introduce new vibrational bands. Similar to 0-Cr, infinitesimal peaks possibly from carbonates and residual water were observed in all Cr-containing amesite samples.

**Figure 8:**
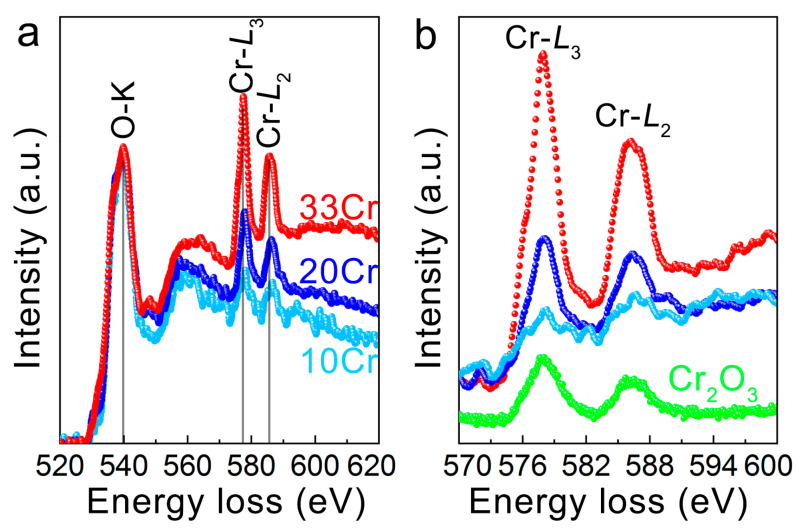
(**a**) EELS of 10Cr, 20Cr and 33Cr showing O-*K*, Cr-*L*_2_ and Cr-*L*_3_ edges. The spectra are rescaled to ensure that the O-*K* edges have the same intensities. Cr-*L*_2_ and Cr-*L*_3_ edges are zoomed in and plotted together with Cr_2_O_3_ in (**b**).

Cr in minerals typically has one of two oxidation states, Cr^3+^ or Cr^6+^ [44]. These oxidation states can be distinguished at the Cr *L*_2,3_ absorption edge because the *L*_3_ and *L*_2_ edges for Cr^6+^ both have two separate peaks, while only one broad peak with weak shoulder peaks is observed for Cr^3+^ [45]. Our previous EELS work demonstrated that Cr in natural lizardite has a trivalent charge [21,46], which agrees with the X-ray absorption near-edge structure studies in kaolin, serpentine and mica minerals [24,47,48,49]. Here, EELS were collected in 10Cr, 20Cr and 33Cr and compared with that of Cr_2_O_3_. As expected, the intensity of Cr edges increases with Cr concentration (Figure 8). Only one separate peak is observed for *L*_3_ (557.8 eV) and *L*_2_ (586.1 eV), implying that Cr in 10Cr, 20Cr and 33Cr is trivalent, as strongly supported by comparing with the edges of Cr_2_O_3_.

Large-quantity substitution of Cr^3+^ in serpentine indicates that Cr^3+^ can be readily used to tune the local strain and acidity of serpentine minerals, which greatly enriches our toolbox to engineer serpentine minerals for various catalytic applications. There remain many motivations for exploring the synthesis of metal-substituted clay minerals. First, clay minerals have applications in nanotechnology and further studies may consider Al^3+^ substitution with other trivalent cations with high catalytic activity [50], like Co^3+^, Fe^3+^, Rh^3+^, etc. Second, extensively substituting Al^3+^ with heavier cations like Cr^3+^ is also helpful to better understand the links between metal substitutions and polytypism in serpentinites. Presently, it is not possible to distinguish Al from Mg and Si by atomic-scale imaging techniques like annular dark-field scanning transmission electron microscopy (ADF-STEM) because of their very close atomic number. Alternatively, we can substitute Al with Cr or other heavy but chemically similar trivalent cations and reveal the structural features by ADF-STEM.

## 4. Conclusions

In summary, we synthesized amesite nanopowders via the hydrothermal method, where the pH of the solution should be controlled at around 12.7 to avoid the formation of impurities like kaolinite and AlOOH. The lamellar amesite particles are 50~100 nm in lateral directions and 10~20 nm in thickness and have abundant stacking defects. Cr can substitute up to ~39.5% Al and is trivalent in Cr-substituted amesite samples. As expected, the crystal lattices expand as Cr concentration increases. Cr substitution has little influence on the polytypism; both endmember and Cr-substituted amesite samples have multiple polytypes, including 6R_2_, 2M_1_, and possibly 2H_2_.

## Figures and Tables

**Table 1:** The conditions for hydrothermal syntheses of Cr-free amesite and the main phase components.

Sample	Gel	Liquid	pH	Products
0Cr-a	1 g	15 mL H_2_O	6.32	kaolinite, AlOOH
0Cr-b	1 g	7.5 mL H_2_O, 7.5 mL 1 M KOH	9.84	amesite, AlOOH
0Cr-c	1 g	5.0 mL H_2_O, 10.0 mL 1 M KOH	12.52	amesite, AlOOH
0Cr-d	1 g	2.5 mL H_2_O, 12.5 mL 1 M KOH	12.72	amesite

**Table 2:** Absorption bands observed in 0Cr.

Bands (cm^−1^)	Interpretation
604	In-plane movement of H atoms
666	Vibration of Si–O
679	Vibration of Si–O
706	Vibration of Si–O
787	Vibration of Si–O
825	Vibration of Si–O
924	Equatorial stretching modes of Si–O
973	Symmetric stretching of apical Si–O
3407	Stretching of the outer hydroxyl group
3606	Stretching of the inner hydroxyl group

**Table 3:** Synthesis conditions, measured Cr-for-Al substitutions and lattice parameters of Cr-containing synthetic amesite. For simplicity, the lattice parameters are presented with the hexagonal structure, adopted by the observed 6R_2_ polytype.

Sample	Gel and Liquid	Al Substitution by Cr	Lattice Parameters (Å)
10Cr	1 g gel, 2.5 mL H_2_O, 12.5 mL 1 M KOH	13.9%	a = 4.596, c = 42.66
20Cr	0.8 g gel, 2.5 mL H_2_O, 12.5 mL 1 M KOH	27.2%	a = 4.623, c = 42.90
33Cr	0.8 g gel, 2.5 mL H_2_O, 12.5 mL 1 M KOH	39.1%	a = 4.629, c = 43.20
For 0Cr, a = 4.59 Å, c = 42.54 Å			

## Data Availability

The original contributions presented in this study are included in the article. Further inquiries can be directed to the corresponding authors.

## References

[1] Dedzo G.K., Detellier C. (2018). Clay Minerals—Ionic Liquids, Nanoarchitectures, and Applications. Adv. Funct. Mater..

[2] Stöter M., Rosenfeldt S., Breu J. (2015). Tunable Exfoliation of Synthetic Clays. Annu. Rev. Mater. Res..

[3] Evans B.W., Hattori K., Baronnet A. (2013). Serpentinite: What, Why, Where?. Elements.

[4] Brigatti M.F., Galan E., Theng B.K.G., Bergaya F., Theng B.K.G., Lagaly G. (2006). Chapter 2 Structures and Mineralogy of Clay Minerals. Developments in Clay Science.

[5] Carmignano O., Vieira S., Brandão P.R., Bertoli A., Lago R. (2020). Serpentinites: Mineral Structure, Properties and Technological Applications. J. Braz. Chem. Soc..

[6] Zhang J., Zhou C.H., Petit S., Zhang H. (2019). Hectorite: Synthesis, Modification, Assembly and Applications. Appl. Clay Sci..

[7] Carniato F., Gatti G., Bisio C. (2020). An Overview of the Recent Synthesis and Functionalization Methods of Saponite Clay. New J. Chem..

[8] Kwak S., Yoo J.-C., Moon D.H., Baek K. (2018). Role of Clay Minerals on Reduction of Cr(VI). Geoderma.

[9] Zhou S., Howard E.S., Liu J., Bashian N.H., Nolan K., Krishnamoorthy S., Rangel G.M., Sougrati M.-T., Prakash G.K.S., Page K. (2017). Hydrothermal Preparation, Crystal Chemistry, and Redox Properties of Iron Muscovite Clay. ACS Appl. Mater. Interfaces.

[10] Sugiura M., Sueyoshi M., Seike R., Hayashi T., Okada T. (2020). Hydrated Silicate Layer Formation on Mica-Type Crystals. Langmuir.

[11] Ji X., Kang Y., Ouyang J., Chen Y., Artzi D., Zeng X., Xiao Y., Feng C., Qi B., Kim N.Y. (2019). Synthesis of Ultrathin Biotite Nanosheets as an Intelligent Theranostic Platform for Combination Cancer Therapy. Adv. Sci..

[12] Joussein E., Petit S., Churchman J., Theng B., Righi D., Delvaux B. (2005). Halloysite Clay Minerals—A Review. Clay Miner..

[13] Cao C.-Y., Liang C.-H., Yin Y., Du L.-Y. (2017). Thermal Activation of Serpentine for Adsorption of Cadmium. J. Hazard. Mater..

[14] Fatnassi M., Solterbeck C.-H., Es-Souni M. (2014). Clay Nanomaterial Thin Film Electrodes for Electrochemical Energy Storage Applications. RSC Adv..

[15] Mellini M., Zanazzi P.F. (1987). Crystal Structures of Lizardite-1T and Lizardite-2H1 from Coli, Italy. Am. Mineral..

[16] Brigatti M.F., Galli E., Medici L., Poppi L. (1997). Crystal Structure Refinement of Aluminian Lizardite-2H_2_. Am. Mineral..

[17] Anderson C.S., Bailey S.W. (1981). A New Cation Ordering Pattern in Amesite-2H_2_. Am. Mineral..

[18] Mackenzie K.J.D., Bowden M.E. (1983). Thermal and Mössbauer Studies of Iron-Containing Hydrous Silicates. IV. Amesite. Thermochim. Acta.

[19] Wiewiora A., Rausell-Colom J.A., Garcia-Gonzalez M.T. (1991). The Crystal Structure of Amesite from Mount Sobotka: A Nonstandard Polytype. Am. Mineral..

[20] Zheng H., Bailey S.W. (1997). Refinement of an Amesite-2H_1_ Polytype from Postmasburg, South Africa. Clays Clay Miner..

[21] Zhang H., Zarzycki P., Gilbert B., Banfield J.F. (2022). Polytypism in Semi-Disordered Lizardite and Amesite by Low-Dose HAADF-STEM. Am. Mineral..

[22] Oze C., Fendorf S., Bird D.K., Coleman R.G. (2004). Chromium Geochemistry of Serpentine Soils. Int. Geol. Rev..

[23] Oze C., Fendorf S., Bird D.K., Coleman R.G. (2004). Chromium Geochemistry in Serpentinized Ultramafic Rocks and Serpentine Soils from the Franciscan Complex of California. Am. J. Sci..

[24] Mitsis I., Godelitsas A., Göttlicher J., Steininger R., Gamaletsos P.N., Perraki M., Abad-Ortega M.M., Stamatakis M. (2018). Chromium-Bearing Clays in Altered Ophiolitic Rocks from Crommyonia (Soussaki) Volcanic Area, Attica, Greece. Appl. Clay Sci..

[25] Maksimović Z., White J.L., Logar M. (1981). Chromium-Bearing Dickite and Chromium-Bearing Kaolinite from Teslić, Yugoslavia. Clays Clay Miner..

[26] Maksimovic Z., Brindley G.W. (1980). Hydrothermal Alteration of a Serpentinite near Takovo, Yugoslavia, to Chromium-Bearing Illite/Smectite, Kaolinite, Tosudite, and Halloysite. Clays Clay Miner..

[27] Khoury H.N., Al-Zoubi A.S. (2014). Origin and Characteristics of Cr-Smectite from Suweileh Area, Jordan. Appl. Clay Sci..

[28] Dzene L., Brendlé J., Limousy L., Dutournié P., Martin C., Michau N. (2018). Synthesis of Iron-Rich Tri-Octahedral Clay Minerals: A Review. Appl. Clay Sci..

[29] Zhou C.H., Zhou Q., Wu Q.Q., Petit S., Jiang X.C., Xia S.T., Li C.S., Yu W.H. (2019). Modification, Hybridization and Applications of Saponite: An Overview. Appl. Clay Sci..

[30] Petit S., Baron F., Decarreau A. (2017). Synthesis of Nontronite and Other Fe-Rich Smectites: A Critical Review. Clay Miner..

[31] Zhang D., Zhou C.-H., Lin C.-X., Tong D.-S., Yu W.-H. (2010). Synthesis of Clay Minerals. Appl. Clay Sci..

[32] Bentabol M., Ruiz Cruz M.D. (2013). Chemistry, Morphology and Structural Characteristics of Synthetic Al–Ni and Al–Co-Lizardites. Appl. Clay Sci..

[33] Huertas F.J., Huertas F., Linares J. (1993). Hydrothermal Synthesis of Kaolinite: Method and Characterization of Synthetic Materials. Appl. Clay Sci..

[34] Bentabol M., Ruiz Cruz M.D., Huertas F.J. (2009). Hydrothermal Synthesis (200 °C) of Co–Kaolinite and Al–Co–Serpentine. Appl. Clay Sci..

[35] Hall S.H., Bailey S.W. (1979). Cation Ordering Pattern in Amesite. Clays Clay Miner..

[36] Bailey S.W. (1969). Polytypism of Trioctahedral 1:1 Layer Silicates. Clays Clay Miner..

[37] Serna C.J., Velde B., White J.L. (1977). Infrared Evidence of Order-Disorder in Amesites. Am. Mineral..

[38] Balan E., Saitta A.M., Mauri F., Lemaire C., Guyot F. (2002). First-Principles Calculation of the Infrared Spectrum of Lizardite. Am. Mineral..

[39] Fuchs Y., Linares J., Mellini M. (1998). Mössbauer and Infrared Spectrometry of Lizardite-1T from Monte Fico, Elba. Phys. Chem. Miner..

[40] Elgayyar T., Azzolina-Jury F., Thibault-Starzyk F. (2025). Infrared spectroscopy at the surface of carbonates. Phys. Chem. Chem. Phys..

[41] Cheng H., Frost R.L., Yang J., Liu Q., He J. (2010). Infrared and infrared emission spectroscopic study of typical chinese kaolinite and halloysite. Spectrochim. Acta Part A Mol. Biomol. Spectrosc..

[42] Dódony I., Pósfai M., Buseck P.R. (2002). Revised Structure Models for Antigorite: An HRTEM Study. Am. Mineral..

[43] Wang Y., Ohishi Y., Shishido T., Zhang Q., Yang W., Guo Q., Wan H., Takehira K. (2003). Characterizations and catalytic properties of Cr-MCM-41 prepared by direct hydrothermal synthesis and template-ion exchange. J. Catal..

[44] Weckhuysen B.M., Wachs I.E., Schoonheydt R.A. (1996). Surface Chemistry and Spectroscopy of Chromium in Inorganic Oxides. Chem. Rev..

[45] Garvie L.A.J., Craven A.J., Brydson R. (1994). Use of Electron-Energy Loss Near-Edge Fine Structure in the Study of Minerals. Am. Mineral..

[46] Zhang H., Gilbert B., Banfield J.F. (2021). Atomic Perspective on the Serpentine–Chlorite Solid-State Transformation. Chem. Mater..

[47] Cardelli A., Cibin G., Benfatto M., Della Longa S., Brigatti M.F., Marcelli A. (2003). A Crystal-Chemical Investigation of Cr Substitution in Muscovite by XANES Spectroscopy. Phys. Chem. Miner..

[48] Brigatti M.F., Galli E., Medici L., Poppi L., Cibin G., Marcelli A., Mottana A. (2001). Chromium-Containing Muscovite: Crystal Chemistry and XANES Spectroscopy. Eur. J. Mineral..

[49] Balan E., Allard T., Morin G., Calas G. (2002). Incorporation of Cr^3+^ in Dickite: A Spectroscopic Study. Phys. Chem. Miner..

[50] Yang G., Zhou L. (2020). Montmorillonite-Catalyzed Conversions of Carbon Dioxide to Formic Acid: Active Site, Competitive Mechanisms, Influence Factors and Origin of High Catalytic Efficiency. J. Colloid Interface Sci..

